# Undiagnosed congenital adrenal hyperplasia in a young woman: MRI insights into complex genital malformations

**DOI:** 10.1016/j.radcr.2025.04.031

**Published:** 2025-05-02

**Authors:** Sofia Bianchi, Maria Gloria Angeretti, Valeria Molinelli, Filippo Piacentino, Massimo Venturini

**Affiliations:** a Vita-Salute San Raffaele University, Via Olgettina, Milan 58 – 20132, Italy; b Diagnostic and Interventional Radiology Department, Circolo and Fondazione Macchi Hospital, ASST Sette Laghi, Viale Borri, Varese 57 – 21100, Italy; c University of Insubria, Via Ottorino Rossi, Varese 9 – 21100, Italy

**Keywords:** Congenital adrenal hyperplasia (CAH), Ambiguous genitalia, Magnetic resonance imaging (MRI), Virilization, Pseudohermaphroditism, Sexual dysfunction

## Abstract

A 23-year-old female patient was referred to the radiology department for evaluation of sexual dysfunction. A previous gynecological examination revealed clitoral hypertrophy and a vagina explorable to a depth of only 2 cm. Hormonal testing showed reduced cortisol levels, along with elevated ACTH and testosterone. Cytogenetic analysis confirmed a 46, XX karyotype; FISH testing was negative for the SRY gene. Pelvic MRI revealed clitoromegaly with evidence of corpora cavernosa forming a penis, a small prostate in the periurethral region, and a vagina that converged with the urethra, forming a common urogenital sinus. No structures suggestive of a scrotum or soft tissue indicative of testicles were observed. The uterus and ovaries appeared normal. Abdominal MRI demonstrated right adrenal hyperplasia.

Congenital adrenal hyperplasia (CAH) is a group of autosomal recessive disorders resulting from defects in adrenal steroidogenesis, with affected individuals displaying various genital malformations. MRI plays a key role in diagnosing and evaluating these complex genital malformations, both of internal and external genitalia. It aids in addressing symptoms of affected individuals, such as recurrent urinary infections, urinary retention, and sexual dysfunction, and provides preoperative imaging prior to surgical correction. MRI also enables the detection of a prostate gland in patients with CAH, serving as a valuable tool for prostate cancer screening when combined with PSA monitoring.

## Introduction

Congenital adrenal hyperplasia (CAH) is a group of autosomal recessive disorders caused by gene mutations encoding enzymes critical for cortisol biosynthesis [1]. Historically, the condition was known as “adrenogenital syndrome” due to the presentation of ambiguous genitalia. The diagnosis of CAH is classically based on a thorough examination of the external genitalia at birth, alongside screening blood tests. However, genital ambiguity is typically absent in males and may only present mildly in females, leading to missed diagnoses of classical CAH during the neonatal period [2].

The case presented in this report outlines the diagnostic process of pseudohermaphroditism in a young woman who, unusually, did not receive a prenatal or neonatal diagnosis of congenital adrenal hyperplasia. This case is particularly significant as, in the present day, it is rare for congenital adrenal hyperplasia to remain undiagnosed in the prenatal or postnatal period or to go untreated until adulthood. In contrast to gynecological ultrasound, magnetic resonance imaging (MRI) is a noninvasive imaging modality that provides a detailed assessment of internal and external genital anomalies. For this reason, MRI should be considered the gold standard for diagnosing and preoperative planning in cases of CAH with complex genital malformations.

## Case presentation

A 23-year-old female patient was referred to the radiology department for assessment of sexual dysfunction. Her familial, medical, and surgical history were unremarkable; she was not receiving any pharmacological treatment. Menarche occurred at the age of 14, with her last menstruation 2 to 3 months prior. She had not undergone any prior gynecological diagnostic examinations and had never had a Pap smear. The patient presented to the gynecologist with difficulty in sexual intercourse, having had no prior symptoms or awareness of any underlying condition. A gynecological examination revealed clitoral hypertrophy, and the vagina, later better identified as a common urogenital sinus, was palpable to a depth of only 2 cm. Transvaginal ultrasound could not be performed due to technical difficulties. Transabdominal ultrasound revealed a uterus of normal shape, volume, and echostructure, with a regular endometrium. The ovaries were unremarkable, with no evidence of masses, and no fluid accumulation was observed in the Douglas pouch. An endocrinological consultation and an MRI of the lower abdomen were recommended. Hormonal testing demonstrated reduced cortisol levels and elevated levels of adrenocorticotropic hormone (ACTH), testosterone, androstenedione, and 17-hydroxyprogesterone (Table 1). The blood levels of follicle-stimulating hormone, luteinizing hormone, 17-beta estradiol, dehydroepiandrosterone sulfate, prostate-specific antigen, and creatinine were normal (Table 1). Blood and urinary electrolytes were also analyzed. No clinically significant alterations were observed, except for mild hypocalciuria (Table 2).

**Table 1:** Hormone values.

Hormone	Value	Unit of measurement	Normal value
FSH	7.0	mUI/mL	Follicular phase: 3.5-12.5 Ovulatory phase: 4.7-21.5 Luteal phase: 1.7-7.7
LH	1.8	mUI/mL	Follicular phase: 2.4-12.6 Ovulatory phase: 14-95.6 Luteal phase: 1.0-11.4
17-beta estradiol	31.90	pg/mL	Follicular phase: 12.5-166 Ovulatory phase: 85.8-498 Luteal phase: 43.8-211
Cortisol	3.34	mg/dL	6.24-18.00
ACTH	247.0	pg/mL	3.6-60.5
Testosterone	3.43	ng/mL	0.05-0.47
DHEAS	196.00	mg/dL	148.00-407.00
Androstenedione	5.50	ng/mL	0.40-4.10
17-hydroxyprogesterone	20.00	ng/mL	0.00-2.85
PSA	0.524	ng/mL	0.00-2.50
Creatinine	1.03	mg/dL	0.50-0.90

ACTH, Adrenocorticotropic hormone; DHEAS, Dehydroepiandrosterone sulfate; FSH, Follicle-stimulating hormone; LH, Luteinizing hormone; PSA, Prostate-specific antigen.

**Table 2:** Electrolytes values.

Electrolytes	Value	Unit of measurement	Normal value
Sodium	139	mEq/l	133-146
Potassium	4.47	mEq/l	3.60-5.30
Chloride	101	mEq/l	96-113
Magnesium	1.91	mg/dl	1.58-2.55
Calcium	10.26	mg/dl	8.50-10.50
Phosphorus	3.41	mg/dl	2.70-4.50
Urine Sodium	197.00	mEq/24h	50-200
Urine Calcium	85.7	mg/24h	100.0-300.0
Urine Potassium	84	mEq/24h	30-90

**Fig. 1:**
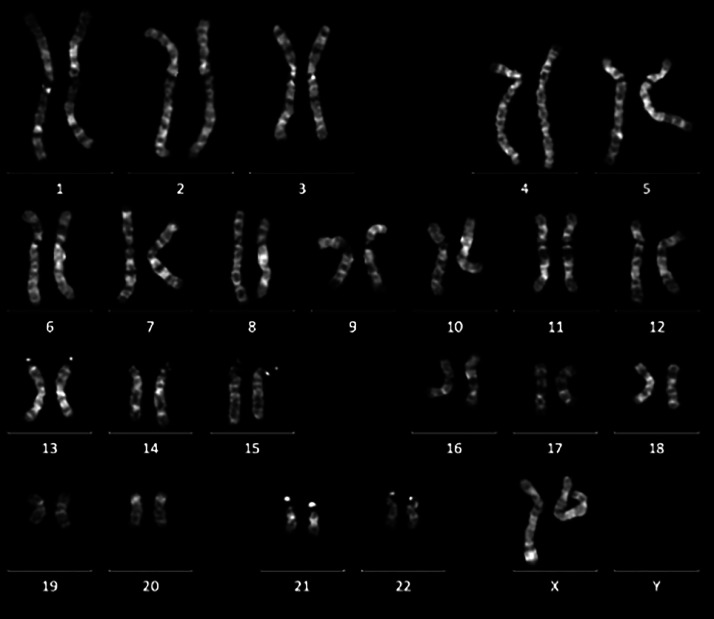
Cytogenetic analysis showing a normal female karyotype (46, XX).

**Fig. 2:**
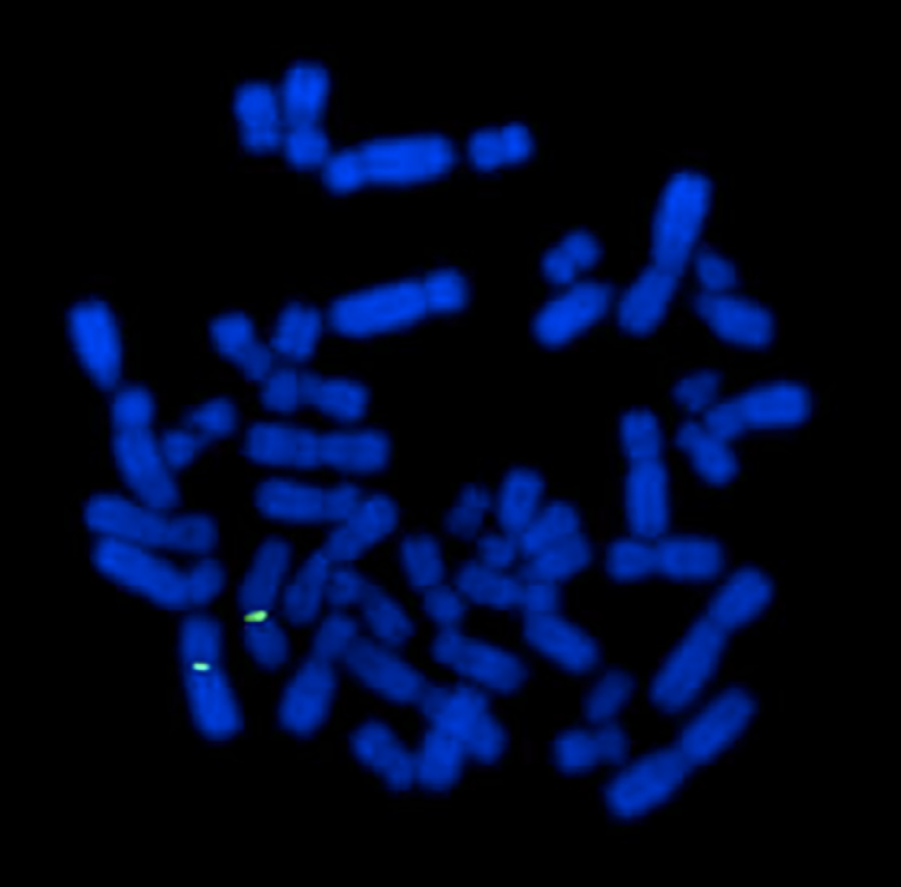
Fluorescence in situ hybridization (FISH) showing a negative hybridization pattern for the probe specific to the SRY gene. Probes are nucleotide sequences complementary to the target nucleotide sequence. Two probes were used: a probe for the region of interest (SRY gene–Yp11.3) (red probe), and a control probe (green probe) mapping to the centromere of the X chromosome (Xp11.1–q11.1). No red signal is observed, indicating the absence of the SRY gene.

Cytogenetic analysis showed a normal female karyotype (46, XX) (Fig. 1), and fluorescence in situ hybridization (FISH) testing was negative for the SRY gene (Fig. 2). FISH analysis was performed to exclude the differential diagnosis of a 46, XX SRY-positive disorder of sex development, a condition resulting from an unequal crossover between the Y and X chromosomes during male gametogenesis, leading to the presence of the SRY gene (Sex-determining Region of the Y chromosome) on the X chromosome. Normally located on the short arm of the Y chromosome (Yp11.3), SRY-gene is the critical factor for testis determination. During embryonic development, it initiates the differentiation of the bipotential gonad into testis [3].

**Fig. 3:**
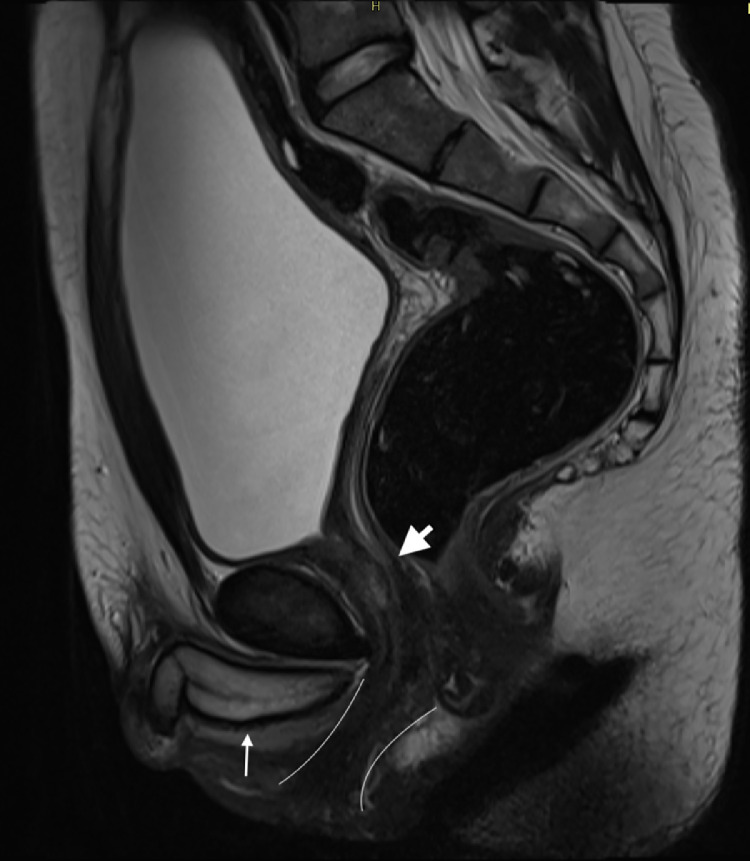
Sagittal T2-weighted MR image shows a hypertrophied clitoris with corpora cavernosa forming a penis (thin arrow), periurethral prostatic tissue (arrowhead), and a common urogenital sinus (outlined by curved lines).

**Fig. 4:**
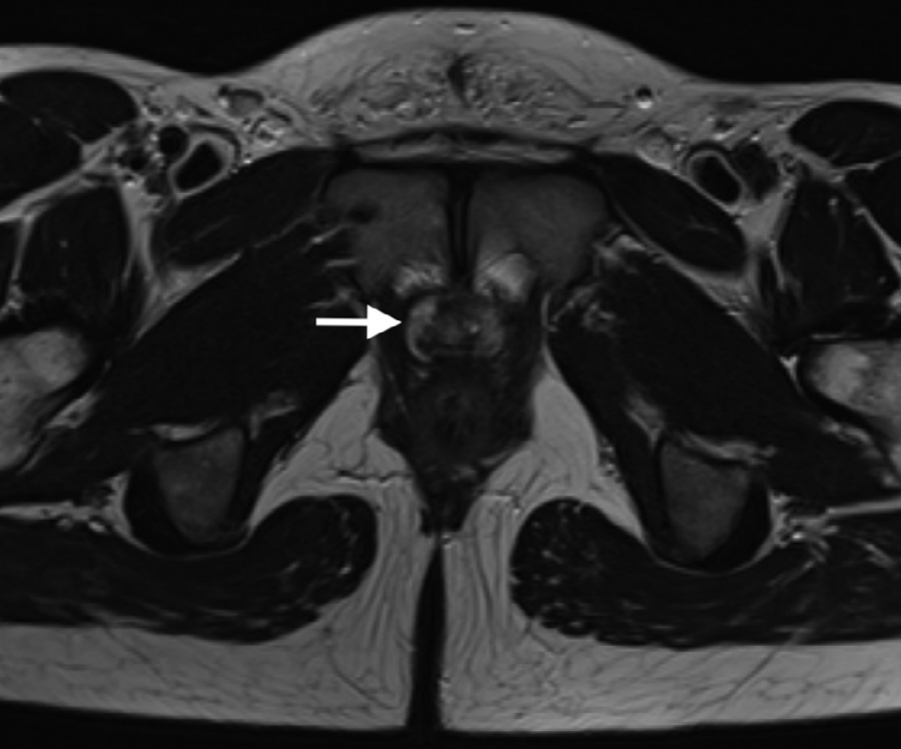
Axial T2-weighted MR image shows periurethral prostate tissue (arrow) with evidence of a hypointense transitional zone and a hyperintense peripheral zone.

**Fig. 5:**
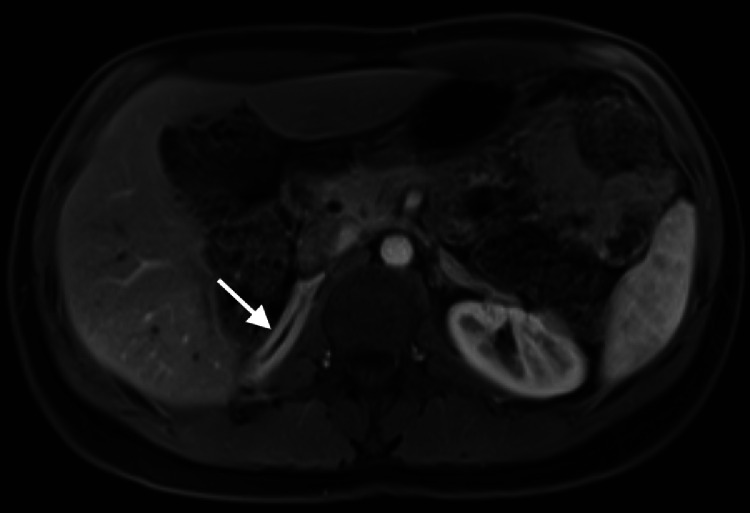
Axial postcontrast-T1 MR image shows homogeneous thickening of the right adrenal gland consistent with adrenal hyperplasia (arrow).

Pelvic MRI confirmed severe hypertrophy of the clitoris, with evidence of developed corpora cavernosa (Fig. 3), resulting in the formation of a penis. A small prostate was identified in the periurethral region, located inferior to the bladder base. On T2-weighted sequences, the prostate exhibited characteristic MR features, including a centrally located, nonhomogeneous, hypointense area corresponding to the central and transitional zones, and a peripheral hyperintense area corresponding to the peripheral zone (Fig. 4). The vagina was observed to converge with the urethra, forming a common urogenital sinus (Fig. 3). The uterus and ovaries displayed normal morphology, with no evidence of structural abnormalities. No scrotal structures or soft tissue indicative of testicular tissue were identified. Abdominal MRI demonstrated right adrenal hyperplasia (Fig. 5).

After diagnosis, the patient began corticosteroid replacement therapy. Moreover, the patient underwent a surgical intervention, specifically a vaginoplasty, which resulted in a positive outcome, improving both functional and aesthetic aspects. Follow-up, including monitoring hormonal values, will be essential to ensure the patient's long-term well-being and fertility.

## Discussion

Congenital Adrenal Hyperplasia (CAH) is a group of autosomal recessive conditions caused by defects in adrenal steroidogenesis. The most common form is due to a deficiency of 21-alfa-hydroxylase, caused by a mutation of the CYP21 gene, which is involved in the biosynthetic pathways of cortisol and aldosterone. The lack of cortisol production leads to compensatory oversecretion of pituitary ACTH and increased levels of cortisol and aldosterone precursors. These excess precursors are converted into testosterone, leading to high blood levels. In the classical form, affected individuals exhibit virilization of the external genitalia, while the internal genitalia (i.e., the uterus and fallopian tubes) are typically normal, and the gonads are female. The late-onset form presents with signs of virilization, such as hirsutism, acne, and oligomenorrhea, which may be mistaken for polycystic ovary syndrome. The definitive diagnosis is established through mutational analysis of the responsible gene. The CYP21 gene is located on the short arm of chromosome 6 (6p21.3), adjacent to the nonfunctional pseudogene CYP21P. Sequence differences between the 2 genes are minimal but sufficient to inactivate CYP21P. Most mutations causing the adrenogenital syndrome arise from recombinations between CYP21 and CYP21P, with majority involving gene conversion mechanisms that transfer deleterious mutations to CYP21. In some cases, recombination results in partial deletion of CYP21, leading to its inactivation, while a small percentage of cases are due to point mutations in CYP21. This variety of mutations explains the variable clinical presentation of the syndrome. Mutations that abolish the enzymatic activity of 21-alfa-hydroxylase generally lead to the classical form, while mutations with residual enzymatic activity result in the virilizing form [3]. Even within the classical form, the extent of genital malformation can vary. In 1954, Prader classified 5 types of genital malformations in patients with CAH [[4], [5], [6]]:

- Type 1: female external genitalia with clitoromegaly.
- Type 2: clitoromegaly with partial labial fusion forming a funnel-shaped urogenital sinus.
- Type 3: clitoromegaly with complete labioscrotal fusion forming a single opening urogenital sinus.
- Type 4: complete scrotal fusion with the urogenital sinus opening at the base of the penis.
- Type 5: complete masculinization of the genitalia, with a normally formed penis and scrotum.

In our case, the presence of the urogenital sinus opening at the base of the penis is consistent with a type 4 malformation.

Appropriate medical and surgical treatment enables affected individuals to achieve good fertility outcomes. Standard medical therapy involves corticosteroid replacement. Surgical correction in patients with CAH aims to achieve 2 primary objectives: to restore a normal appearance of the external genitalia – genitoplasty – and to separate the vagina from the common urogenital sinus – vaginoplasty [4]. The timing of surgical intervention remains a topic of debate. Initially, it was thought that complete correction could be achieved with a single procedure in early childhood. However, later studies demonstrated that the aesthetic outcomes of these single-stage procedures were often unsatisfactory, with most patients requiring additional surgeries [7,8]. As a result, it has been suggested that definitive vaginoplasty may be best postponed until after puberty [4].

MRI plays a key role not only in diagnosing CAH but also in evaluating complex genital malformations of both the internal and external genitalia. It provides essential preoperative imaging before surgical correction, and helps address symptoms in affected patients, such as recurrent urinary infections due to the common urogenital sinus, inability to engage in typical sexual intercourse due to vaginal anomalies, or urinary retention due to benign prostatic hyperplasia. The paraurethral Skene’s glands surrounding the female urethra are homologous to the prostate gland in males. Both structures originate from the urogenital sinus during embryonic development. This common origin explains the anatomical and functional similarities between the 2 glands. These glands are lined by pseudostratified, mucin-positive columnar epithelium and contain prostate-specific antigen (PSA) [9]. The prostate in males and Skene's glands in females are both involved in the production of specific secretions, although the function of these secretions varies. In males, the prostate plays a key role in semen production and ejaculation, while in females, the Skene’s glands are primarily thought to contribute to lubrication. To date, there have been 17 reported cases of prostatic tissue development in female CAH patients [10]. The identification of a prostate gland is significant, as it carries the potential for both benign prostatic hyperplasia and prostate cancer [9,11,12]. MRI is a valuable tool for screening for prostate cancer, particularly when combining with PSA monitoring.

## Conclusion

CAH is a complex condition that requires careful diagnosis and management, combining hormonal testing, genetic analysis, and advanced imaging techniques. MRI plays a crucial role in both the diagnosis and preoperative assessment of CAH, providing detailed images of the complex genital malformations in affected patients. It helps address symptoms in affected patients, and identify potential complications, such as the presence of prostatic tissue in female CAH patients, which requires close monitoring for conditions like benign prostatic hyperplasia and prostate cancer.

## Patient consent

Written informed consent was obtained from the patient for publication of this case report, including any accompanying images. A copy of the signed consent form is available for review by the Editor-in-Chief of this journal upon request.
